# Supplementary material for: Length-dependent disassembly maintains four different flagellar lengths in Giardia
Source: eLife. 2019 Dec 19;8:e48694. doi: 10.7554/eLife.48694 (PMC6992383; doi:10.7554/eLife.48694)
Supplement: Transparent reporting form [file elife-48694-transrepform.pdf]

eLIFE
1st Floor
24 Hills Road
Cambridge CB2 1JP, UK
P 01223 855340
W elifesciences.org
T @elife

eLife's transparent reporting form

We encourage authors to provide detailed information *within their submission* to facilitate the interpretation and replication of experiments. If you have any questions, please contact us: editorial@elifesciences.org.

Sample-size estimation

- You should state whether an appropriate sample size was computed when the study was being designed
- You should state the statistical method of sample size computation and any required assumptions
- If no explicit power analysis was used, you should describe how you decided what sample (replicate) size (number) to use

Please outline where this information can be found within the submission (e.g., page numbers or figure legends), or explain why this information doesn't apply to your submission:

Effect sizes were not pre-specified due to the exploratory nature of our flagellar length measurements and IFT particle tracking in a multiciliated organism. No less than 30 fluorescent traces were used to draw conclusions for kymograph experiments of IFT particle tracking. For all fluorescence intensity measurements, at least 10 cells from three biological replicates were used where at least two flagella are measurable in the same cell.

Replicates

- You should report how often each experiment was performed
- You should include a definition of biological versus technical replication
- The data obtained should be provided and sufficient information should be provided to indicate the number of independent biological and/or technical replicates
- If you encountered any outliers, you should describe how these were handled
- Criteria for exclusion/inclusion of data should be clearly stated
- High-throughput sequence data should be uploaded before submission, with a private link for reviewers provided (these are available from both GEO and ArrayExpress)

Please outline where this information can be found within the submission (e.g., page numbers or figure legends), or explain why this information doesn't apply to your submission:

eLife Sciences Publications, Ltd is a limited liability non-profit non-stock corporation incorporated in the State of Delaware, USA, with company number 5030732, and is registered in the UK with company number FC030576 and branch number BR015634 at the address 1st Floor, 24 Hills Road, Cambridge CB2 1JP | August 2014

The number of biological replicates is described in figure legends and methods, and each experiment was performed at least three times. Over 35 length measurements were performed for each flagellar pair (Methods). Of the four flagellar pairs, length measurements were limited three pairs (caudal, anterior, posteriolateral) to due to difficulties in length measurements for ventral flagellar pair.

Statistical reporting

- Statistical analysis methods should be described and justified
- Raw data should be presented in figures whenever informative to do so (typically when N per group is less than 10)
- For each experiment, you should identify the statistical tests used, exact values of N, definitions of center, methods of multiple test correction, and dispersion and precision measures (e.g., mean, median, SD, SEM, confidence intervals; and, for the major substantive results, a measure of effect size (e.g., Pearson's r, Cohen's d)
- Report exact p-values wherever possible alongside the summary statistics and 95% confidence intervals. These should be reported for all key questions and not only when the p-value is less than 0.05.

Please outline where this information can be found within the submission (e.g., page numbers or figure legends), or explain why this information doesn't apply to your submission:

Total N, p, and statistical test definitions are included in the main text and figure legends. Our N values are typically well above 10 for IFT imaging experiments and over 35 for flagellar length measurements. Flagellar lengths and IFT particle tracking was scored between pairs within the same cell and pairs also scored between cells. Statistical methods for FRAP and fluorescence intensity experiments are detailed in the Methods section.

(For large datasets, or papers with a very large number of statistical tests, you may upload a single table file with tests, Ns, etc., with reference to page numbers in the manuscript.)

Additional data files ("source data")

- We encourage you to upload relevant additional data files, such as numerical data that are represented as a graph in a figure, or as a summary table
- Where provided, these should be in the most useful format, and they can be uploaded as "Source data" files linked to a main figure or table
- Include model definition files including the full list of parameters used
- Include code used for data analysis (e.g., R, MatLab)
- Avoid stating that data files are "available upon request"

Please indicate the figures or tables for which source data files have been provided:

Detailed definitions and equations for flagellar length models are provided in the Results and in the Methods section. Custom python script measurements for mean intensities and custom plots are deposited in GitHub.

eLife Sciences Publications, Ltd is a limited liability non-profit non-stock corporation incorporated in the State of Delaware, USA, with company number 5030732, and is registered in the UK with company number FC030576 and branch number BR015634 at the address 1st Floor, 24 Hills Road, Cambridge CB2 1JP | August 2014
